# *PRNP *genetic variability and molecular typing of natural goat scrapie isolates in a high number of infected flocks

**DOI:** 10.1186/1297-9716-42-104

**Published:** 2011-09-30

**Authors:** Eirini G Fragkiadaki, Gabriele Vaccari, Loukia V Ekateriniadou, Umberto Agrimi, Nektarios D Giadinis, Barbara Chiappini, Elena Esposito, Michela Conte, Romolo Nonno

**Affiliations:** 1 National Agricultural Research Foundation, Veterinary Research Institute, Thessaloniki, Greece; 2 Istituto Superiore di Sanità, Department of Veterinary Public Health and Food Safety, Rome, Italy; 3 Aristotle University of Thessaloniki, Faculty of Veterinary Medicine, Clinic of Productive Animal Medicine, Thessaloniki, Greece

## Abstract

One hundred and four scrapie positive and 77 negative goats from 34 Greek mixed flocks were analysed by prion protein gene sequencing and 17 caprine scrapie isolates from 11 flocks were submitted to molecular isolate typing. For the first time, the protective *S146 *variant was reported in Greece, while the protective *K222 *variant was detected in negative but also in five scrapie positive goats from heavily infected flocks. By immunoblotting six isolates, including two goat flockmates carrying the *K*222 variant, showed molecular features slightly different from all other Greek and Italian isolates co-analysed, possibly suggesting the presence of different scrapie strains in Greece.

## Introduction, Methods and Results

Scrapie is a prion disease that affects sheep and goats. It belongs to the group of transmissible spongiform encephalopathies (TSE) that also comprises the zoonotic bovine spongiform encephalopathy (BSE). Major determinants for sheep scrapie occurrence have been demonstrated to be the host susceptibility, regulated mainly by the prion protein encoding gene (*PRNP*) [1,2] and the prion strain [3]. However, the association of genetic variability of goat *PRNP *with resistance to TSE is still under investigation [4,5] and limited knowledge is available on natural goat prion strains [4,6-8]. Greece has the largest goat population in Europe [5], mainly bred in mixed flocks, and the second higher prevalence of goat scrapie (175 scrapie positive goats over 40 034 tested during 2002-2008) [9]. In this study *PRNP *coding sequence of Greek goats, from both scrapie affected and healthy goats was investigated, in regards to their gene variability. However, due to the lack of appropriate sampling of negative goats, this work is not a case control study. Molecular strain typing by discriminatory western blotting (WB) was also implemented on ~10% of the scrapie positive goats detected between 2002 and 2008.

Among samples diagnosed at the Greek National Reference Laboratory for TSE, by using TeSeE BIORAD and TeSeE Western Blotting BIORAD, archived frozen obex tissues from 106 positive (derived from 34 flocks from 13 geographic areas) and 77 negative goats (collected from five flocks with high scrapie prevalence) were submitted to genotyping analysis (Table 1). Most goats were asymptomatic except for three clinical suspects, and one that appeared suspect at slaughter (Table 2). The mean age of scrapie tested goats was around 36 months (ranging from 12 to 86 months).

**Table 1:** *PRNP *genotypes of scrapie positive and negative goats ^1^.

	***PRNP *codon**																	**Number of goats per genotype**	
			
**PrP genotype**	**37**	**54-95**	**110**	**127**	**136**	**142**	**143**	**146**	**151**	**154**	**168**	**173**	**211**	**222**	**239**	**240**		**Scrapie positive**	**Scrapie negative**
		
1	GG	5OR:5OR	TT	GG	AA	II	HH	NN	RR	RR	PP	SS	RR	QQ	SS	PP		44	12
2	GG	5OR:5OR	TT	GG	AA	II	HH	NN	RR	RR	PP	SS	RR	QQ	SS	PS		30	23
3	GG	5OR:5OR	TT	GG	AA	II	HH	NN	RR	RR	PP	SS	RR	QQ	SS	SS		9	3
4	GG	5OR:5OR	TT	GG	AA	ΙΙ	HH	ΝΝ	RR	‡RH	PP	SS	RR	QQ	SS	PS		4	3
5	GG	5OR:5OR	TT	GG	AA	ΙΙ	HH	ΝΝ	RR	‡RH	PP	SS	RR	QQ	SS	SS		2	2
6	GG	5OR:5OR	TT	GG	AA	II	HH	NN	RR	RR	PP	SS	RR	‡QK	SS	PS		1	2
7	GG	5OR:5OR	TT	‡GS	AA	‡IM	HH	NN	RR	RR	PP	SS	RR	QQ	SS	PP		1	1
8	GG	*5OR:4OR	TT	GG	AA	II	HH	NN	RR	RR	PP	SS	RR	QQ	SS	PP		3	
9	GG	5OR:5OR	‡TP	GG	AA	II	HH	NN	RR	RR	PP	SS	RR	‡QK	SS	SS		3	
10	GG	5OR:5OR	TT	GG	†AT	II	HH	NN	RR	RR	PP	SS	RR	QQ	SS	SS		1	
11	GG	5OR:5OR	TT	GG	AA	II	HH	NN	RR	†RQ	PP	SS	RR	QQ	SS	PP		1	
12	GG	5OR:5OR	TT	GG	AA	ΙΙ	HH	ΝΝ	‡RH	RR	PP	SS	RR	QQ	SS	PS		1	
13	GG	5OR:5OR	TT	GG	AA	II	HH	NN	RR	RR	‡QQ	SS	RR	QQ	SS	PP		1	
14	GG	5OR:5OR	TT	‡GS	AA	II	HH	NN	RR	‡RH	PP	SS	RR	QQ	SS	PS		1	
15	GG	5OR:5OR	TT	GG	AA	‡IM	HH	NN	RR	RR	PP	SS	RR	QQ	SS	PS		1	
16	GG	5OR:5OR	TT	GG	AA	II	HH	NN	RR	RR	PP	SS	RR	‡QK	SS	SS		1	
17	GG	5OR:5OR	‡TP	GG	AA	ΙΙ	HH	ΝΝ	RR	RR	PP	SS	RR	QQ	SS	PS			5
18	GG	5OR:5OR	TT	GG	AA	II	HH	NN	RR	RR	‡PQ	SS	RR	QQ	SS	PS			3
19	GG	5OR:5OR	TT	GG	AA	II	HH	‡NS	RR	RR	PP	SS	RR	QQ	SS	PP			2
20	GG	5OR:5OR	TT	GG	AA	II	HH	‡NS	RR	RR	PP	SS	RR	QQ	SS	PS			2
21	GG	5OR:5OR	TT	GG	AA	II	HH	NN	RR	RR	‡PQ	SS	RR	QQ	SS	PP			2
22	GG	5OR:5OR	TT	‡GS	AA	II	HH	NN	RR	RR	PP	SS	RR	QQ	SS	PP			2
23	GG	5OR:5OR	TT	GG	AA	ΙΙ	HH	ΝΝ	RR	RR	PP	SS	‡RQ	‡QK	SS	SS			1
24	GG	*5OR:4OR	TT	GG	AA	II	HH	NN	RR	RR	PP	SS	RR	QQ	SS	PS			1
25	GG	5OR:5OR	TT	GG	AA	II	‡HR	NN	RR	‡RH	PP	SS	RR	QQ	SS	PS			1
26	GG	5OR:5OR	TT	GG	AA	II	‡HR	NN	RR	RR	PP	SS	RR	QQ	SS	PP			1
27	GG	5OR:5OR	TT	GG	AA	II	‡HR	NN	RR	RR	PP	SS	RR	QQ	SS	PS			1
28	GG	5OR:5OR	‡TP	GG	AA	ΙΙ	HH	ΝΝ	RR	RR	‡PQ	SS	RR	QQ	SS	PS			1
29	GG	5OR:5OR	TT	GG	AA	ΙΙ	HH	ΝΝ	RR	RR	PP	†SN	RR	QQ	SS	PP			1
30	GG	5OR:5OR	TT	GG	AA	II	HH	NN	RR	RR	PP	SS	RR	QQ	†FF	SS			1
31	GG	5OR:5OR	TT	GG	AA	II	HH	NN	RR	RR	‡PQ	‡SN	RR	QQ	SS	PP			1
32	GG	5OR:5OR	TT	GG	AA	II	HH	‡NS	RR	RR	‡PQ	SS	RR	QQ	SS	PP			1
33	GG	5OR:5OR	TT	GG	AA	II	HH	‡SS	RR	RR	PP	SS	RR	QQ	SS	PP			1
34	‡GV	5OR:5OR	TT	GG	AA	II	HH	NN	RR	RR	PP	SS	RR	QQ	SS	SS			1
35	‡GV	5OR:5OR	TT	‡GS	AA	II	HH	NN	RR	RR	PP	SS	RR	QQ	SS	PS			1
36	‡GV	5OR:5OR	TT	‡GS	AA	II	HH	NN	RR	RR	PP	SS	RR	QQ	SS	SS			1
37	‡GV	5OR:5OR	TT	GG	AA	II	HH	NN	RR	RR	PP	SS	RR	QQ	SS	PS			1
																	Total (*n*)	104	77

^1^*5OR = five octapeptide repeats, 4OR = four octapeptide repeats, †: novel polymorphisms observed in goats, ‡: *PRNP *polymorphisms differing from the wild-type goat.

**Table 2:** Natural scrapie isolates analysed with discriminatory western blotting by using mAbs P4 and SAF84^2^.

Sample's ID	*PRNP *genotype	Target group	Age (months)	rel ratio	SD	mw	SD	dig	SD	mono	SD	non	SD
GR-L1-G1	*wt, 240P/P*	sl	21	1.21	0.35	17.54	0.17	50	2	31	1	19	3
GR-D1-G1	*wt, 240P/P*	sl	86	0.63	0.19	17.70	0.02	52	1	32	1	16	2
GR-E1-G1	*wt, 168Q/Q, 240P/P*	sl	40										
GR-B1-G1	*wt, 240P/S*	sl	58	1.15	0.41	17.54	0.08	53	2	33	5	14	4
GR-M1-G1	no amplicon	sl	43	1.21	0.21	17.60	0.17	53	1	30	3	17	4
GR-N1-G1	no amplicon	sl	20	1.05	0.09	17.57	0.16	52	2	32	2	17	3
GR-F1-G1	*wt, 240P/P*	sl	75	0.97	0.19	17.67	0.10	52	1	30	2	18	4
GR-A9-G1	*wt, 240P/P*	sl	48	1.11	0.12	17.62	0.11	54	1	30	3	16	4
GR-A4-G1	*wt, 240P/S*	sl	36	1.02	0.28	17.59	0.02	55	1	29	2	16	3
GR-A4-G2	*wt, 240P/P*	sl	36	0.93	0.22	17.59	0.12	54	2	30	3	16	4
GR-A4-G3	*wt, 240P/P*	sl	36	0.84	0.09	17.62	0.12	51	3	31	3	17	6
GR-A4-G4	*wt, 240P/P*	sl	24	0.85	0.16	17.61	0.12	52	2	30	2	18	3
GR-A4-G5	*wt, 240P/P*	susp. sl	48	1.10	0.07	17.58	0.11	50	2	31	2	18	3
*GR-A4-S1*	*wt*	sl	-	1.01	0.13	17.59	0.10	49	2	34	3	16	4
GR-A11-G1	*wt, 240P/P*	susp. flock	24	0.61	0.12	17.72	0.03	52	2	32	2	16	5
GR-A11-G2	*wt, 240P/S*	susp. flock	26	0.58	0.08	17.82	0.10	53	4	29	3	18	4
*GR-A11-S1*	*wt*	susp. flock	9	0.41	0.01	17.96	0.10	48	2	31	0	21	1
*GR-A11-S2*	*ARQ/TRQ*	susp. flock	9	0.41	0.02	17.92	0.06	48	4	32	1	20	3
GR-A2-G1	*wt, 222Q/K, 240S/S*	sl	36	0.38	0.01	18.03	0.07	48	2	35	1	17	1
GR-A2-G2	*wt, 222Q/K, 240S/P*	sl	36	0.50	0.03	18.04	0.02	48	2	33	2	19	4
IT1-goat	*wt, 240P/S*	-	-	0.98	0.05	17.56	0.11	48	2	31	3	20	3
IT2-goat	*wt, 240P/S*	-	-	0.90	0.03	17.52	0.07	49	2	34	3	17	4
IT3-goat	*wt, 240P/S*	-	-	0.87	0.17	17.51	0,04	53	3	30	2	17	1
IT4-goat	*wt, 240P/P*	-	-	1.05	0.07	17.56	0.06	52	4	29	1	19	3
*IT5-sheep*	*wt*	-	-	1.00		17.59	0.07	52	3	31	2	17	3
*sheep BSE*	*wt*	exp	-	8.58	1.85	16.78	0.07	71	4	20	2	9	3

^2 ^Sample's ID indicates the country of origin (GR for Greece, IT for Italy), the geographic area of origin (alphabetical letters), the flock from that area (i.e. A1) and the number of the goat (G) or sheep (*S, in italics*)-cases from the same flock (G1), i.e. GR-A1-G1. Abbreviations: wt: *ARQ/ARQ *wild type, sl: slaughtered, susp.: TSE suspect in, exp: experimentally infected, rel ratio: relative ratio, SD: standard deviation, mw: molecular weight, dig: di-glycosylated PrP^res^, mono: mono-glycosylated PrP^res^, non: non-glycosylated PrP^res^.

Genotyping was performed as previously described [10,11]. Two samples gave no PCR amplification and the analysis of the remaining 104 samples revealed 37 *PRNP *genotypes (Table 1). Twenty polymorphic codons and an octapeptide deletion polymorphism were detected. Sixteen polymorphisms resulted into amino acid substitutions at codons *G37V, T110P, G127S, A136T, I142M, H143R, N146S, R151H, R154H/Q, P168Q, S173N, R211Q, Q222K, S239F, S240P*, while 5 were silent nucleotide polymorphisms (codons 42 *cca*→*cc****g***; 138 *agc*→*ag****t***, 231 *agg *→***c****gg*, 237 *ctc*→*ct****g ***and 238 *ttt*→*tt****c***). In 177 out of 181 goats, five octapeptide repeats between codons 54-95 were observed, while in four goats (3 scrapie-positive and one scrapie-negative) a 24*bp *deletion was detected between codons 70-77, resulting in four octapeptide repeats [12] instead of the usual three or five repeats reported in goats [13]. For the first time, codon 136 was observed as polymorphic in the goat. Interestingly, this polymorphism, *A136T*, is the same observed very rarely in sheep in Greece [14]. It is noteworthy that an additional novel polymorphism in goats, *R154Q*, was observed. These two polymorphisms were observed in single scrapie-positive goats. Two novel amino-acid polymorphisms were also observed in negative goats, *S173N *and *S239F*. The *S146 *variant, associated with scrapie protection in Cyprus [12,15], was observed only in negative goats from two different flocks. The *K222 *variant [10,16-18] was observed in five scrapie affected animals derived from two independent outbreaks and from two different scrapie endemic regions (A and J prefecture) (see Additional File 1); negative flockmates were unavailable. This variant was also found in negative goats, deriving from two of the five flocks analysed. Nucleotide polymorphisms at codons 21, 23, 49, 171 and 220 already reported in Greek goats [7,8,14,18] were not observed during this survey.

Seventeen of the above caprine isolates originating from 11 affected flocks were tested by discriminatory immunoblotting, along with sheep flockmates from heavily affected mixed flocks i.e. one sheep from A4 and two from A11 (Table 2). Samples were selected with respect to available tissue for WB analysis and to be more or less representative of the country's whole territory. The isolates were from regions with high (flocks A2, A4, A9, A11, J1), intermediate (flocks L1, D1, E1, B1, F1) and low (flocks M1, N1) scrapie incidence (Table 2) (see Additional file 1). Four Italian caprine isolates from four different flocks, an Italian sheep scrapie isolate and a sheep BSE sample were also included in the study.

Molecular typing of proteinase K resistant PrP (PrP^res^) from positive cases was performed by ISS discriminatory WB [19] using the monoclonal antibodies SAF84 (residues 163-173 of ovine PrP; Spi-Bio, Montigny Le Bretonneux, France) and P4 (residues 89-104 of ovine PrP; RIDASCREEN, R-Biopharm, Germany). In each blot, an Italian sheep scrapie isolate and a sheep experimentally infected with BSE [20] were used as internal controls. The principle of discrimination of this testing strategy is based on the different N-terminal cleavage for proteinase K digestion of PrP between sheep BSE and scrapie [21-26] which results in different apparent molecular mass of scrapie and BSE PrP^res ^and in the partial loss of the N-terminal amino acid sequence WGQGGSH in sheep BSE samples. For each sample, apparent molecular mass, SAF84/P4 ratio relative to the scrapie control and glycoform ratio were obtained from at least 3 independent determinations.

**Figure 1:**
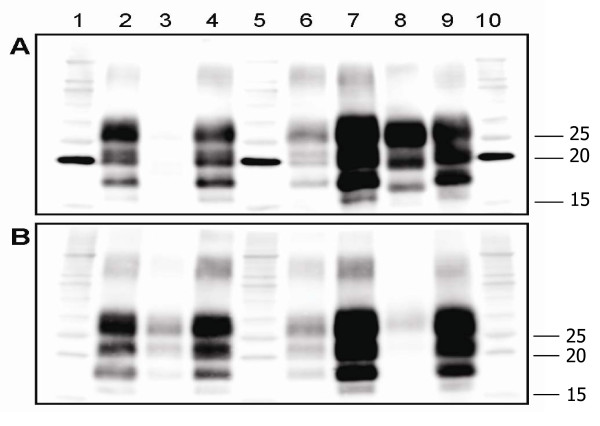
**Discriminatory WB of Greek scrapie positive goats**. WB of proteinase K-treated PrP^res ^from the obex of Greek goats (GR-A4-G2, GR-E1-G1, GR-A4-G3 and GR-B1-G1 loaded in lanes 2-4 and 6, respectively), an Italian goat (IT1-goat, lane 7), a sheep BSE (lane 8) and an Italian sheep used as internal control (IT5-sheep, lane 9). PrP^res ^was detected by mAbs SAF84 (panel A) or P4 (panel B) using molecular markers (lanes 1, 5, 10). Note that sample GR-E1-G1 (lane 3) is negative with SAF84 and positive with P4 and this is probably due to P168Q polymorphism within the SAF84 epitope.

**Figure 2:**
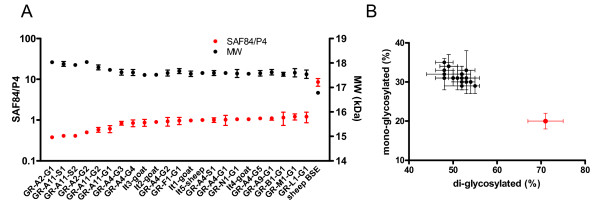
**Comparison of PrP^res ^molecular mass, SAF84/P4 ratio and glycoform ratio**. **A**: apparent molecular mass (black symbols, right y axis, linear scale) and SAF84/P4 relative ratio (red symbols, left y axis, log scale) of all scrapie samples analysed in comparison to sheep BSE. Samples are ordered from the lowest to the highest SAF84/P4 ratio. Sheep BSE displays the highest SAF84/P4 ratios and the lowest apparent molecular mass. Note that samples with the lowest SAF84/P4 ratios also display the highest apparent molecular masses and that these samples include Greek sheep and goat deriving from the same A11 flock (see Table 1). **B**: Scattergraph of proportions of proteinase K-treated di-glycosylated and mono-glycosylated PrP^res ^in scrapie (black symbols) and sheep BSE (red symbol). Error bars represent standard deviations of three independent determinations.

In immunoblots, caprine isolates showed apparent molecular masses and SAF84/P4 ratios similar to the scrapie control (Table 2, Figure 1). The mean apparent molecular mass (± SD) for sheep BSE was 16.78 ± 0.07 kDa, well lower than that observed for scrapie samples (from 17.51 ± 0.04 kDa to 18.04 ± 0.02 kDa) (Table 2, Figure 2a). Similarly, the relative SAF84/P4 ratio for sheep BSE was 8.58 ± 1.85, while it was comprised between 0.38 ± 0.01 and 1.21 ± 0.35 in scrapie. The glycoform ratio also allowed a clear-cut discrimination of sheep BSE from all scrapie isolates (Figure 2b). Scrapie samples, indeed, were characterised by a lower ratio (from 48:31 to 55:29) than sheep BSE (71:20). Among scrapie samples, some Greek isolates showed a slightly higher apparent molecular mass and a slightly lower SAF84/P4 ratio compared to the other Greek and Italian isolates (Figure 2a). The six isolates, showing the highest apparent molecular mass of PrP^res ^and the lowest SAF84/P4 ratio, were two goats carrying *K222 *variant from flock A2 (GR-A2-G1 and -G2) and two sheep (GR-A11-S1 and -S2) and two goats (GR-A11-G1 and -G2) from flock A11 (Table 2, Figure 2a). When compared to all other scrapie isolates, these six scrapie cases showed a significantly higher apparent molecular mass (17.92 ± 0.05 vs. 17.54 ± 0.04, *P *= 0.0002) and a significantly lower SAF84/P4 ratio (0.48 ± 0.04 vs. 1.01 ± 0.03, *P *< 0.0001).

Noteworthy, a caprine sample (GR-E1-G1) did not react with SAF84, although it was positive with P4 (Figure 1). After PrP^res ^concentration, this sample was barely positive with SAF84 and strongly positive with P4 (data not shown). This prevented us from determining its molecular parameters (Table 2). Given that sample GR-E1-G1 was homozygous for Q at codon 168, which is comprised in the SAF84 epitope, we hypothesised that *Q168 *might hamper binding by SAF84. Indeed, another C-terminal mAb, L42, whose epitope does not include the amino acid residue 168, gave a clear scrapie positive immunoblot pattern (data not shown).

## Discussion

In the present survey, extensive genotyping of scrapie positive goats from Greece and molecular isolate typing of caprine cases from regions with various prevalence of scrapie was performed. Since the present study is not a case control study, no clear association of the PrP genotype with scrapie susceptibility can be implemented, given that no flock data about the frequencies of *K222 *and other alleles are available. Our findings demonstrate however a high *PRNP *genetic variability in Greek goats and the presence of novel nucleotide polymorphisms at codons 136 (*A136T*), 154 (*R154Q*), 173 (*S173N*) and 239 (*S239F*). Moreover for the first time, the presence of *N146S *polymorphism in Greek scrapie negative goats was observed.

The observation of positive *K222 *carriers in scrapie endemic areas was of particular interest. The cases were derived from three heavily scrapie infected flocks (A2, A4 and J1). Unfortunately, due to lack of flock data, we could not investigate whether *K222 *carriers were subject to a strong infectious pressure in flocks with high scrapie prevalence or they were simply not protected from scrapie infection, possibly due to the involvement of a scrapie strain different from those previously studied in Italy and France [10,16,17] or Greece [18].

The ISS discriminatory WB method allowed a clear-cut biochemical discrimination of Greek goat scrapie isolates from BSE. The biochemical data of GR-E1-G1 sample brought us to conclude that *Q168 *homozygosis inhibits the antibody binding probably because of the SAF84 paratope-epitope mismatch. On this point, the polymorphic epitopes in goat *PRNP *gene might reveal a potential negative impact on the performances of diagnostic and discriminatory testing of goat scrapie where anti-PrP antibodies are used [27,28].

Interestingly, most of the Greek isolates showed molecular characteristics identical to Italian goat scrapie cases, while isolates from two outbreaks deriving from regions with high scrapie prevalence (flocks A2 and A11), including positive goats bearing the *K222 *variant, showed slight but statistically significant molecular differences compared to the other Greek and Italian isolates. Whether these molecular differences are indicative of the presence in those outbreaks of a different scrapie strain remains to be determined. The biological characterization of Greek isolates by bioassays in transgenic mice and bank voles is currently underway.

## List of Abbreviations

TSE: transmissible spongiform encephalopathies; BSE: bovine spongiform encephalopathy; PrP: prion protein; *PRNP*: prion protein encoding gene; WB: western blotting; PrP^res ^: proteinase K resistant prion protein

## Competing interests

The authors declare that they have no competing interests.

## Authors' contributions

EL, FE, NR, VG, AU conceived the study, participated in its design and coordination. FE, VG, RN, LE helped to draft the manuscript. NG participated in the design of the study. FE, VG, CM, CB carried out the molecular genetic studies and participated in the sequence alignment. NR, EE, FE carried out the immunoblotting. All authors read and approved the final manuscript.

## Supplementary Material

Additional file 1 **Geographic location of the scrapie positive goat flocks (number in parenthesis) tested per prefecture (capital letters)**. The number in parenthesis indicates the total number of flocks tested per prefecture (i.e. A (15) means 15 flocks tested from A region). Click here for file
