# Supplementary figures and images for: PRNP genetic variability and molecular typing of natural goat scrapie isolates in a high number of infected flocks
Source: Vet Res. 2011 Sep 30;42(1):104. doi: 10.1186/1297-9716-42-104 (PMC3190342; doi:10.1186/1297-9716-42-104)

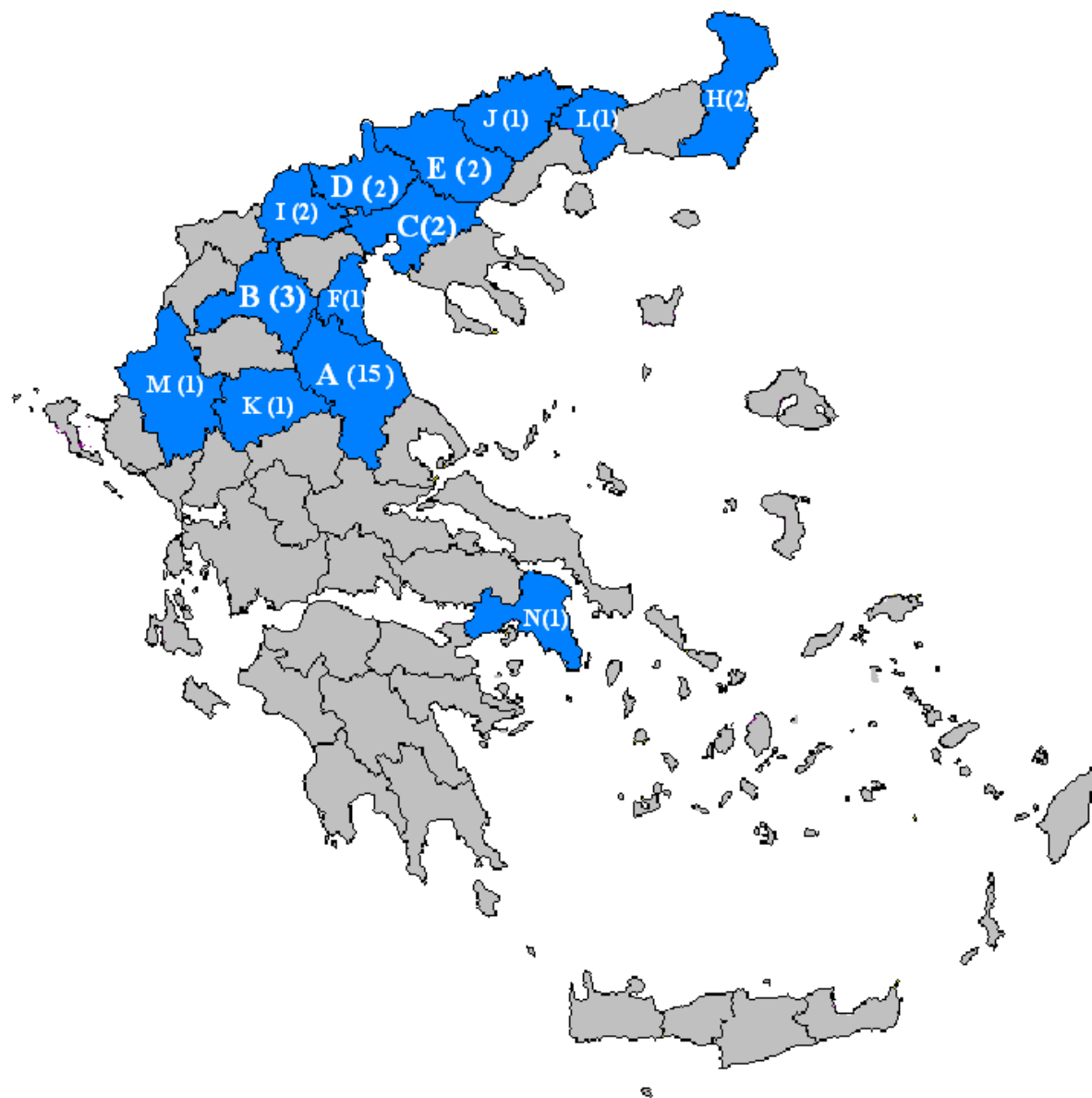

Supplement: Additional file 1 — Geographic location of the scrapie positive goat flocks (number in parenthesis) tested per prefecture (capital letters). The number in parenthesis indicates the total number of flocks tested per prefecture (i.e. A (15) means 15 flocks tested from A region). [file 1297-9716-42-104-S1.PDF]
